# Pregnancy after drug-free in vitro activation of follicles and fresh tissue autotransplantation in primary ovarian insufficiency patient: a case report and literature review

**DOI:** 10.1186/s13048-018-0447-3

**Published:** 2018-08-31

**Authors:** F. Fabregues, J. Ferreri, J.M. Calafell, V. Moreno, A. Borrás, D. Manau, F. Carmona

**Affiliations:** 1 Institut Clinic of Gynecology, Obstetrics and Neonatology (ICGON), Barcelona, Spain; 2 Hospital Clinic, Barcelona, Spain; 3 Institut d’Investigacions Biomèdiques August Pi I Sunyer (IDIBAPS), Barcelona, Spain

**Keywords:** Primary ovarian insufficiency, In vitro activation, Akt stimulation, Hippo signaling

## Abstract

**Background:**

The aim of this report was to describe a case of pregnancy after drug-free in vitro activation (IVA) of follicles and fresh tissue autotransplantation in primary ovarian insufficiency (POI) patient and to review the pertinent literature.

**Methods:**

We present a case in wich a 32 - years old patient with POI became pregnant after IVA without tissue culture and with ovarian tissue transplantation. We also reviewed the literature using Pubmed database.

**Case presentation:**

Pretreatment with estradiol/progesterone stopped the day before surgery. The removal of the ovarian cortex and autotransplantation were performed by laparoscopy in the same surgical act. Ovarian fragments were transplanted in contralateral ovary and peritoneal pocket near to the ovary. Immediately after surgery GnRH agonist together HMG injections started, leading the growth of 3 preovulatory follicles and the retrieval of two mature eggs. After IVF two embryos were transferred and singleton pregnancy was established and currently she is 25 weeks pregnant.

**Results:**

A total of 51 patients with POI in whom an in vitro activation of ovarian tissue was performed, were collected from the revieew of the literature. In 29.4% of them, follicular development was obtained and in 4 of them a pregnancy**.** In all of them, a combined technique (fragmentation and activation) was performed in two laparoscopies**.** No case has been reported successfully after drug-free in vitro activation.

**Conclusions:**

This is the first report about a case with pregnancy after drug-free in vitro activation of follicles and fresh tissue autotransplantation in POI patient.

## Background

Patients with primary ovarian insufficiency (POI) develop menopausal symptoms at less than 40 years of age. They have few remaining follicles and their only chance for bearing a baby is through egg donation [1]. Recently, has been developed a method for the activation of dormant follicles using in vitro culture of ovarian fragments treated with PI3K stimulators and PTEN inhibitors in mice and human [2]. Subsequent studies suggested that ovarian fragmentation could interfere with the ovarian Hippo signaling pathway, also leading to ovarian follicle growth [3]. Kawamura et al. combined these two methods in an in vitro activation (IVA) approach to treat infertility in patients with POI [4].

It has been suggested that the IVA approach could be used in patients with diminishing ovarian reserve (DOR) and early stage of POI. Because these patients have spontaneous activation of dormant primordial follicles reaching the secondary stage, secondary follicle growth could be promoted in these patients using drug-free IVA without tissue culture. For patients with ovaries containing residual secondary follicles, it is likely that the fragmentation step (Hippo signaling disruption) alone is sufficient to promote follicle growth [5].

We present the case of a patient with POI in which a pregnancy was achieved using drug-free IVA without tissue culture. After the ovarian tissue transplantation, GnRH agonist and HMG treatment led to the growth three preovulatory follicles, which brought to the retrieval of two oocytes. After IVF two embryos were transferred. The patient is currently 25 weeks pregnant. We also reviewed the literature using the Pubmed database.

## Methods of literature review

We searched the Pubmed database (up to April 2018) using, the key words “primary ovarian insufficiency”, “in vitro activation” and “Akt stimulation”. Our search generated 22 articles, those not written in english were excluded.

Of the remaining articles, reviews and related to animal experimentation were discarded. Finally, 3 articles were selected that will be the ones discussed here that collect all the published cases in which the in vitro activation of ovarian tissue has been applied in patients with premature ovarian failure with their results.

## Case presentation

We are presenting a 32 years old patient whom reached menarche at 11 y of age. At 28 years of age, she experienced irregular cycles and became amenorrheic at 30 years of age with elevated FSH levels (89.9 UI/L) and AMH levels (0.02 ng/ml). Despite diverse testing including chromosome analysis, her pathogenesis was unknown.

The Ethics Research Committee of the Hospital Clinic of Barcelona, Spain approved the treatment, and patient provided written informed consent. In order to supress serum gonadotropin levels before grafting ovarian fragments pretreatment with oral estrogen/progesterone (Progyluton; Bayer, Spain) was performed during one month previously surgery.

We used a laparoscopic approach to extract ovarian tissue. For the removal of the ovarian cortex we use scissors to avoid the use of coagulation to minimize the surgical trauma to the sensitive ovarian tissue. Bipolar coagulation was used on the medulla after extraction of the ovarian cortex and was performed sparingly to protect the remaining ovary. We opted to remove two-thirds of the ovarian cortex. The medulla was removed by dissection with small scissors before thin layers of ovarian cortices (1–2 mm thickness) were, cut into small strips (0.5–1 × 0.5–1 cm, 1–2 mm thickness). At the same time, 10–20% of the volume of each ovarian strip were used for histological analysis to determine the presence of residual follicles. In this patient any residual follicles were found in the ovarian cortex based on histological analyses.

**Fig. 1:**
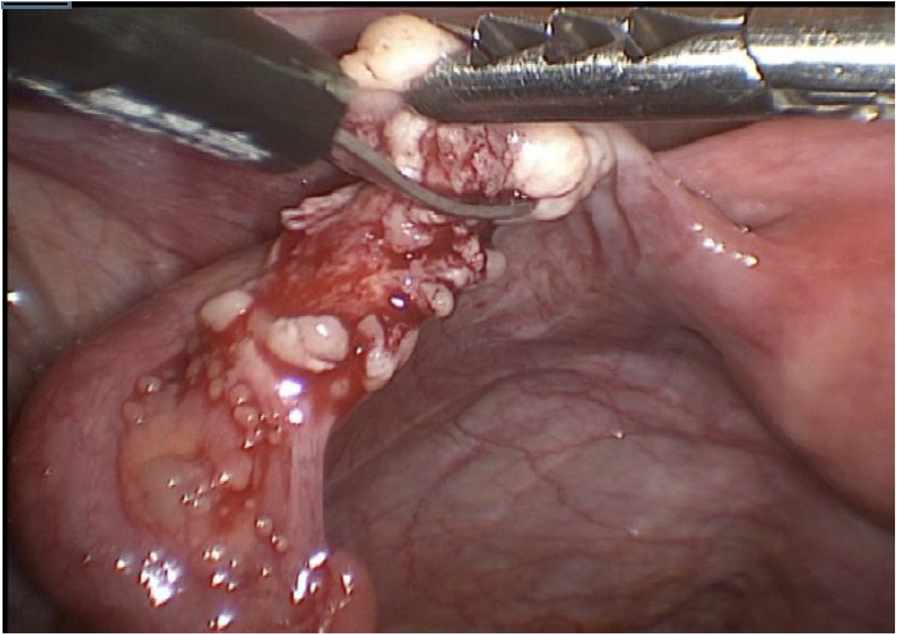
Laparoscopic extraction of ovarian tissue

**Fig. 2:**
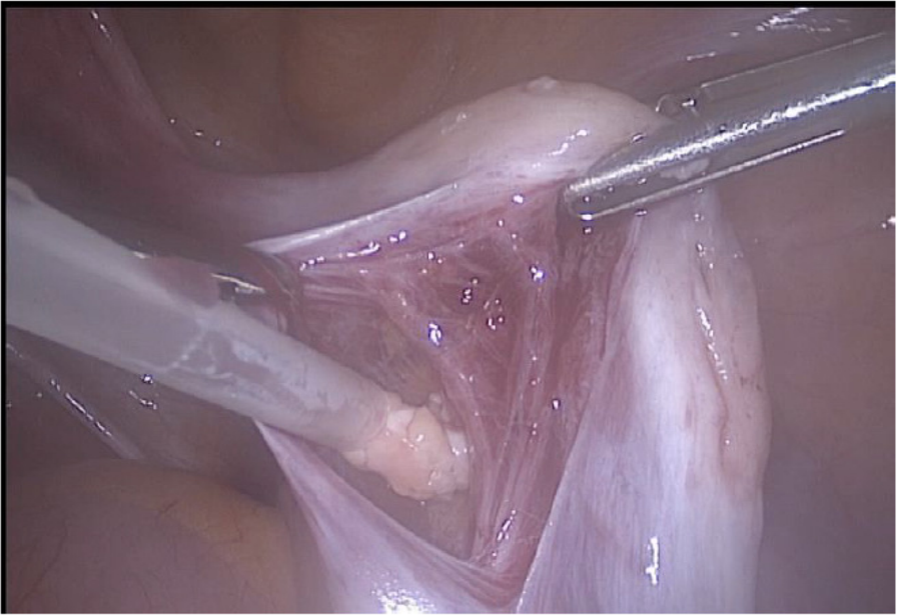
Placement of ovarian fragments in the peritoneal pocket near to the ovary

**Fig. 3:**
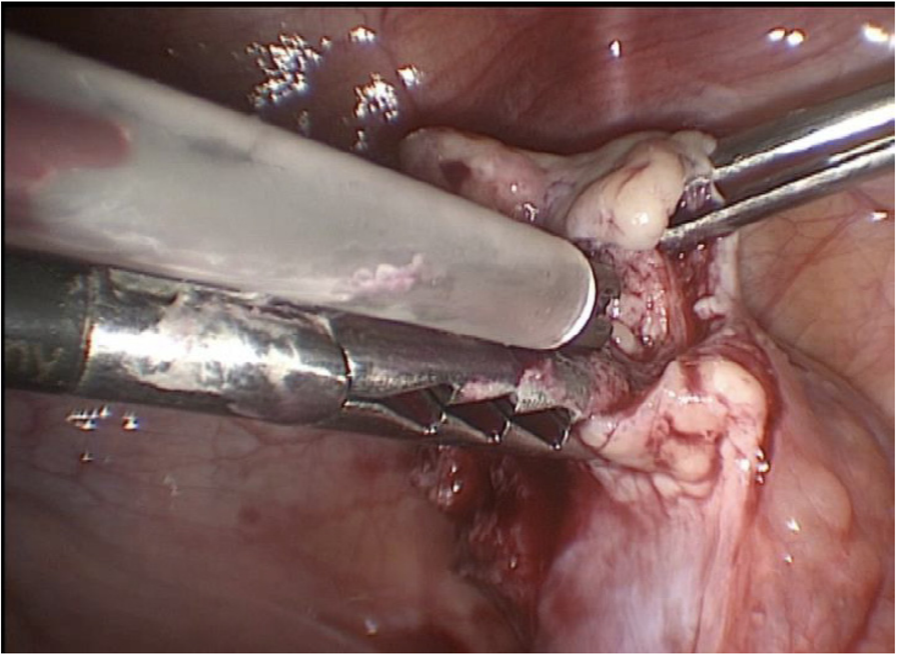
Placement of ovarian fragments in contralateral ovary

**Fig. 4:**
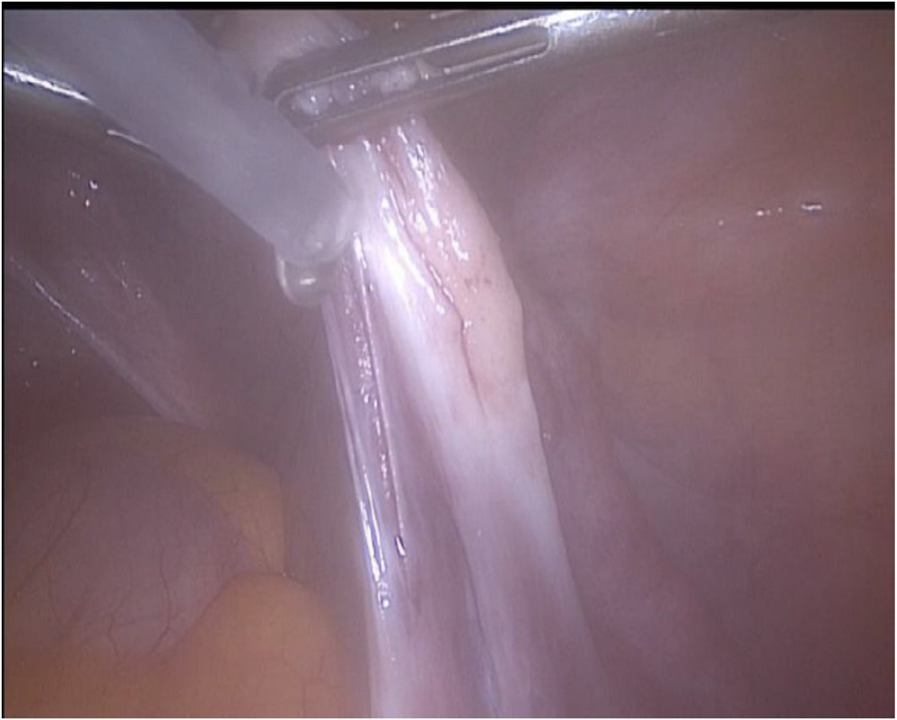
Bonding the edges of peritoneum pocket with N.Hexyl-2- cyanoacrylate.

**Fig. 5:**
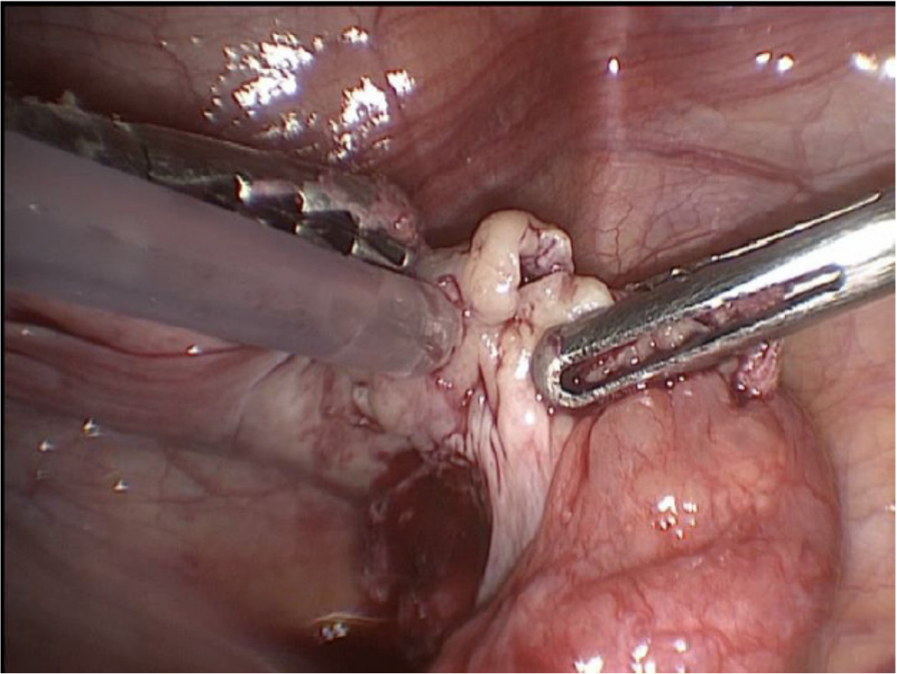
Bonding the edges of contralateral ovary with N.Hexyl-2- cyanoacrylate.

The auto-transplantation was performed by laparoscopy in the same surgical act, and the graft of ovarian tissue was performed in contralateral ovary and peritoneal pocket near to the ovary. The incisions were made with scissors avoiding the use of electric coagulation, depositing 10–12 fragments of ovarian tissue in each of the places described. We use of N hexyl-2 cyanoacrylate as a fixation surgical treatment in the transplantation site during ovarian tissue transplantation in order to avoid sutures [6] (Figs. 1, 2, 3, 4 and 5). The patient was discharged the same day of the surgery without complications. Pretreatment with estradiol/progesterone stopped the day before ovary autografting, inducing withdrawal bleeding soon after the surgery. After initiation of withdrawal bleeding, injection of GnRH agonist (triptorelin, 0.1 mg/d)(Decapetyl; Ipsen Pharma, Spain). was initiated together daily purified urinary HMG (300 IU) (Meriofert; Angelini, Spain).

After 20 days of ovarian stimulation, 3 follicles (18,17, 16 mm) were observed by transvaginal ultrasound in the transplantation site. Recombinant hCG (Ovitrelle 250, Merck, Spain) was administered and thirty-six hours later oocyte retrieval was performed. Two mature oocytes were retrieved, fertilization was achieved through ICSI and day 3, two embryos were transferred. Singleton pregnancy was established and currently she is 25 weeks pregnant. Both, the first trimester ultrasound screening for chromosomal abnormalities, and the analysis of second trimester triple screen for Down syndrome, did not find any abnormalities.

## Results

The results of the literature review are show in Table 1. The data collected in this review correspond to 51 patients with POI who underwent an in vitro activation of ovarian tissue. In all of them, a combined technique (fragmentation and activation) was performed in two laparoscopies. In 15 (29.4%) follicular development was observed with different stimulation protocols, recovering a total of 30 oocytes achieving 4 pregnancies. In three of them a live born was obtained.

**Table 1:** Previously reported cases with IVA in POI

Author/year	Ref.	No. Patients	IVA technique	Patients with follicle development	Total oocytes retrieved	Pregnancies
Kawamura 2013; Suzuki 2015	[4, 16]	37	PI3K activation PTEN inhibition + Hippo signal disruption	9	24	3 (1 miscarriage 2 live births)
Zhai 2016	[18]	14	PI3K activation PTEN inhibition + Hippo signal disruption	6	6	1 (1 live birth)
Our case		1	Hippo signal disruption only	1	2	1 (ongoing)

Although in the first group of 37 patients the follicular response was only observed in those in which there were residual follicles in the remitted material for histological study, in another study the follicular development was also observed in 2 patients without residual primordial follicles.

## Discussion

Ovarian functions decrease with age, characterized by a diminishing number of follicles and menstrual cycle cessation. In patients with primary ovarian insufficiency (POI), early exhaustion of ovarian follicles is evident due to genetic, immunological, iatrogenic or other causes. POI affects 1% of women and is characterized by high circulating FSH levels together with amenorrhea before 40 years of age [7]. These patients are infertile due to a lack of follicle growth and ovulation. Although menstrual cycles cease in these patients, some of them still contain residual small ovarian follicles which do not produce enough circulating estrogens and progesterone to modulate uterine functions. [8]

POI patients have intermittent and unpredictable ovarian functions. Although 5–10% of patients in reported studies have a chance to conceive, only a 1.5% pregnancy rate was found in controlled trials [9]. Therefore, egg donation has been commonly used to treat infertility in POF patients. Although diverse hormone therapies and ovulation induction treatments have been prescribed for POI patients, these infertility treatments have a limited success. Thus, the development of a new method for treating infertility that would enable POI patients has been anticipated in order to conceive using their own eggs.

In recent years, the demand for fertility preservation for oncologic and nononcologic reasons, has increased dramatically. Currently, embryo cryopreservation and mature-oocyte cryopreservation after ovarian stimulation are the only methods for fertility preservation endorsed by the American Society for Reproductive Medicine [10]. Since 2004, when the first pregnancy after ovarian tissue cryopreservation and transplantation (OTCP) was reported, the number of live births has increased to more than 130, showing a logarithmic growth during the last 2 years and highlighting the need to move from experimental studies to widespread clinical application. After reimplantation of ovarian tissue in the pelvic cavity, the ovarian activity is restored in more than 95% of cases. The mean duration of ovarian function after reimplantation is from 4 to 5 years, but the function can persist for up to 7 years, depending on the follicular density at the time of ovarian-tissue cryopreservation. [11]

It has been suggested that after transplantation, the massive recruitment of “trapped” follicles in the residual ovary may be mediated by mechanism via phosphatase and tensin homolog (PTEN) [12]. This obviously prevents an exact knowledge on whether the oocytes that resulted in the pregnancy originated in the transplanted tissue or not, and this is a limitation that we want to acknowledge.

Earlier studies showed that ovary damage promotes follicle growth in PCOS patients who underwent ovarian wedge resection or laparoscopic ovarian laser drilling [13, 14]. Recently, a method has been developed for the activation of dormant follicles using in vitro culture of ovarian fragments treated with PI3K stimulators and PTEN inhibitors (primordial follicle activators) in mice and human [3, 4]. When this method was applied to treat POI patients, it lead to succesfull pregnancies and deliveries, so that these patients could have their own genetic offspring [4, 15–17]. This treatment was repeated in China, also leading to succesful delivery in a POI patient [13]. In addition, in this approach, in vitro activation (IVA), the ovarian cortices containing residual follicles were fragmented into small cubes. Therefore, the fragmentation facilitated the conversion of G-actin into F-actin, leading to disruption of the Hippo signaling pathway to allow secondary follicle growth [4, 11, 18].

Based on the above, it has been suggested that in DOR and early stage of POI patients the spontaneous activations of dormant primordial follicles reaching the secondary follicle stage. The growth of this secondary follicle could be promoted using drug-free IVA without tissue culture. Simply removing the ovarian cortex from these patients, followed by cutting to disrupt ovarian Hippo signaling promoted follicle growth when cortical fragments were grafted back into patients [5]. Taking into account this possibility, we applied this concept in our patient. In fact, given the fast follicular development that ocurred, we should consider an activation of follicles in an advanced stage of growth.

On the other hand, unlike the cases that have been already published, we started an ovarian stimulation with gonadotropins inmediately after the surgery. It is known that follicle development is controlled by local factors and gonadotropins derived from the anterior pituitary. Dependent on the requirement of gonadotropins, follicle development is designated into three stages, gonadotropin-independent: from primordial to primary follicles, gonadotropin-responsible:from primary, secondary to antral follicles, and gonadotropin-dependent: from antral to preovulatory follicle [19]. Earlier studies have demostrated that FSH receptors are expressed in follicles from primary to later stages and, together with the FSH and LH treatment, it promotes preantral follicle growth [10, 20].

Moreover, some local factors as C-type natriuretic peptide (CNP), which is an intraovarian factor important for preantral and antral follicle growth as well as oocyte maturation, are secreted by granullosa cells of secondary and antral follicles in response to FSH stimulation [10, 21]. In our patient, this could explain the ovarian response according to the protocol used.

Although healthy babies have been born, more studies are needed to ensure the safety of the present IVA procedure.

## Conclusions

IVA is effective to promote follicle growth so that POI patients can have their own genetic babies. Nevertheless, for infertile DOR and early stage POI patients, drug-free IVA without tissue culture could offer less invasive treatment by the only disruption of Hippo signal pathway. To our knowledge, this is the first report about a case with pregnancy after drug-free in vitro activation of follicles and fresh tissue autotransplantation in POI patient.

## Abbreviations

AMH: Anti-Müllerian Hormone
CNP: C-type Natriuretic Peptide
DOR: Diminishing Ovarian Reserve
FSH: Follicle-Stimulating Hormone
GnRH: Gonadotropin-Releasing Hormone
hCG: Human Chorionic Gonadotropin
HMG: Human Menopausal Gonadotropin
ICSI: Intracytoplasmic Sperm Injection
IVA: In vitro Activation
LH: Luteinizing Hormone
OTCP: Ovarian tissue cryopreservation and transplantation
PCOS: Polycystic Ovary Syndrome
PI3K: Phosphatidylinositol-4,5-bisphosphate 3-kinase
POI: Primary Ovarian Insufficiency
PTEN: Phosphatase and tensin homolog
